# Some Medico-Legal Points

**Published:** 1908-05-16

**Authors:** 


### Resident Medical Officers' Department.

### SOME MEDICO-LEGAL POINTS.

There are various duties connected with the position of a Resident Medical Officer which are always liable to bring him into relation with legal technicalities and procedures. Some of these are described in text-books of medical jurisprudence, others he frequently has to find out for himself. One of the errors into which newly qualified men not uncommonly fall is in the filling up of death certificates, connected with which there are one or two subtleties. It is, of course, common knowledge that by the " primary " cause of death in a death certificate form is meant the underlying morbid condition of the patient, as, for instance, enteric fever or diabetes, while the space for the " secondary " cause is intended for the more immediate cause of death, such as cardiac failure or acidæmia. It is, perhaps, less well known that all death certificates relating to cases in which accident or violence within a year of death has contributed in any way to that result must give information as to the occurrence of such accident or violence. Great care should always be exercised in filling in certificates for cases of death following operation. There are certain terms which the Registrar-General will accept without cavil, and others, apparently of the same significance, which will ensure return of the certificate and other vexations. Thus post-operative shock and post-operative collapse are held to be on different sides of the Registrar's line, and an eminent surgeon was a year or two ago subpœnaed to attend an inquest because his house surgeon had used the term which is not accepted by the authorities as satisfactory evidence of natural death. Certificates of deaths occurring within one month of delivery should always contain a notice to that effect, whether the parturition has contributed towards the fatal issue or not. Vague terms, a list of which is printed at the beginning of each folio of certificates, should be most carefully avoided, and clear, legible handwriting is everywhere necessary, since the certificate has to be copied by a Registrar who has no professional knowledge.

Some of the most difficult problems of the hospital resident are in connection with patients who have committed penal offences. Thus cases of attempted self-poisoning are common enough, as also are other forms of attempted suicide, as by cutting the throat, gun and pistol shots, and so on. Self-poisoners are, as a matter of fact, comparatively rarely prosecuted by the police, but in all cases of attempted suicide it must be remembered that the resident is certain to be called as a witness if a prosecution should ensue, and he ought to take the most careful notes at the time he admits the patient of everything which may bear upon the case. In cases of attempted suicide brought to hospital otherwise than by the police, the Resident Medical Officer should refrain absolutely from all gossip about the case: his function is not that of a detective, and he must be particularly careful that no indiscreet remark may form the starting point of a criminal charge.

Another case of great difficulty arises when a woman is admitted under circumstances which warrant the belief that she has recently been delivered of a child. It is clearly no business of the Resident Medical Officer to report anything to the police, but the latter not uncommonly apply to the hospitals within easy radius whenever a new-born child is found abandoned under suspicious circumstances. The police will ask whether any woman has been admitted whose condition indicates recent delivery, and such a question places the resident in something of a predicament. If he answers he may expose a patient to a criminal charge; if he does not he is accessory to a felony. The proper line in the circumstances (which are not so very uncommon) is to afford the required information.

It is occasionally the house officer's duty to witness, and even to draw up, a will for some patient *in extremis*, who cannot afford to wait for proper legal assistance. It need scarcely be said that a most careful consideration of the patient's mental state is essential to either proceeding. The writer once witnessed a will drawn up on a sheet of notepaper by a colleague who had luckily had some legal training. The patient was dying of phthisis; but his mind was perfectly clear, and he was desperately anxious to sign his will before it was too late; he died within ten minutes of doing so. Insurance claims the Resident Medical Officer is not often called upon to sign, but whenever he is requested to do so he should always demand a fee; a reasonable charge for this is half a guinea for claims of £50 and under, and twice that sum for higher claims.

At inquests the Resident Medical Officer is a scientific adviser of the court of even more importance than when he appears as an expert witness before other judicial tribunals. In nearly every case the verdict turns chiefly upon his evidence, and the greatest pains to render it clear and simple, beyond the risk of misunderstanding, are never thrown away. The statutory fee for attending an inquest for the ordinary medical man and for hospital residents in cases of death occurring outside the walls of their institution, is one guinea, with an additional guinea for performing a post-mortem examination. For cases, however, which die in hospital, no fee is allowed to any officer of that hospital, as residents quickly discover on taking up their duties.

Anomalies of the law of various other kinds frequently come under the notice of Resident Medical Officers. Thus the darkening of counsel occurring in civil actions in which medical men are subpœnaed by opposite sides to give conflicting testimony is often most extraordinary. Even in the High Court, truth, in spite of its greatness, does not always prevail. Thus not many years ago a carpenter who was the subject of a prepatellar bursa was injured in a street accident. His injuries turned out to be comparatively trivial, and after a stay in hospital of two or three days he was about to be discharged when an energetic house officer offered to relieve him of his bursa. The carpenter gratefully accepted this offer, and was accordingly operated upon. Subsequently he brought an action for damages, and owing to the ignorance and carelessness of the defending barrister actually obtained substantial damages on the ground that his injuries had necessitated a painful operation. No one thought of asking the house officer whether the injury and the operation were in any way connected, and so the jury naturally assumed it. In another case an old lady obtained heavy damages in the High Court for the onset of cataract some months after a minor accident which had dislocated her shoulder. Two medical witnesses were produced in support of her case, and the Hospital Resident who originally treated the dislocation was ignored when he denied any causal relation between the two lesions. From this it will be seen that the best medical opinion will not necessarily be accepted if witnesses can be subpœnaed in superior numbers to contradict it. As long as litigants may pay what they like and subpœna whom they like, so long must the Resident Medical Officer expect to find his pronouncements disregarded now and then by juries and judges, no matter how well founded they may be.

